# Significance of lobular intraepithelial neoplasia at margins of breast conservation specimens: a report of 38 cases and literature review

**DOI:** 10.1186/1746-1596-5-54

**Published:** 2010-08-20

**Authors:** Sophia K Apple, Mahan Matin, Eric P Olsen, Neda A Moatamed

**Affiliations:** 1 Department of Pathology & Laboratory Medicine, David Geffen School of Medicine at UCLA, Los Angeles, California, U.S.A. BOX 951732, 1P-244 CHS Los Angeles, CA 90095-1732 USA

## Abstract

**Background:**

Presence of lobular intraepithelial neoplasia (LIN) is not routinely reported as part of margin assessment in breast conservation therapy (BCT) as in ductal carcinoma in situ (DCIS). With new emerging evidence of LIN as possible precursor lesion, the hypothesis is that LIN at the margin may increase the risk of local recurrence with BCT. The aim is to determine whether there is an increase incidence of recurrence when LIN is found at surgical margins on BCT.

**Methods:**

We retrospectively reviewed a total of 1,334 BCT at a single institution in a 10 year period. Inclusion criteria are positive margin with LIN from primary BCT containing invasive and/or in situ carcinoma with comparison to the negative control group who had similar diseases with negative margin for LIN.

**Results:**

We identified 38 cases (2.8%) with LIN either lobular carcinoma in situ/atypical lobular hyperplasia (LCIS/ALH) at a margin on initial BCT with 36% recurrence rate. Of the 38 cases: 5 (13%) were lost to follow-up, 12 (32%) had no further procedures performed and 21 (55%) had re-excision. Out of 21 patients who had re-excisions, 12 (57%) had residual invasive carcinoma or DCIS, three (14%) had pleomorphic LCIS and 4 (19%) showed residual classic type LCIS. 71% had significant residual disease (local recurrence) and 29% had no residual disease. A negative control group consisted of 38 cases. We found two patients with bone or brain metastasis and one local recurrence. Clinical follow up periods range from 1 to 109 months.

**Conclusions:**

LIN found at a margin on BCT showed a significant recurrent ipsilateral disease. Our study supports the view that LIN seen at the margin may play a role in recurrence.

## Background

Foote and Stewart [1] initially described the entity "lobular carcinoma in situ" (LCIS) in 1941 and described it as a "precancerous" lesion. At that time, a diagnosis of LCIS was treated like a cancer by radical mastectomy. Haagenson et al [2] believed that the term "in situ" was a misnomer and preferred the term "lobular neoplasia" in 1978 because LCIS did not appear as a premalignant lesion but as a marker for increased risk for developing cancer. This notion was based on his observation that only 17% of 211 patients with LCIS developed invasive cancer with a mean follow-up time of 14 years, either ipsilateral and/or contralateral side of the breast. Subsequent studies of LCIS which included epidemiologic data have confirmed that LCIS is a risk factor and not an obligatory precursor lesion as ductal carcinoma in situ (DCIS). There is controversy on the management when lobular neoplasia is found on the core needle biopsy whether to watch carefully or perform an excision. The presence of lobular intraepithelial neoplasia (LIN) at the surgical margin is frequently not reported as part of the pathology analysis as in DCIS or invasive cancer for BCT. When DCIS or invasive carcinoma is found at a surgical margin, re-excision and radiation therapy is the standard management. However, when LIN is found at a surgical margin, it is not routinely reported. No re-excision is done and radiation therapy is not recommended for LIN unless there is a concomitant invasive carcinoma or DCIS in BCT.

Pleomorphic lobular carcinoma in situ (PLCIS) is known as a variant of LCIS which is negative for E-cadherin, supporting lobular differentiation. PLCIS may show central comedo necrosis and a higher nuclear grade resembling DCIS. PLCIS found at a margin has an increased recurrence rate when compared to classic LCIS. Hence it is recommended for re-excision when the surgical margins are involved with PLCIS on BCT. A study on PLCIS at a margin and radiation therapy has yet to be performed and further investigation needs to be done.

Moreover, recent studies show that LCIS and invasive lobular carcinoma (ILC) share a similar genetic molecular biology. New research suggests that there is a genetic progression of LCIS to an invasive carcinoma and that the biologic significance of LCIS may be a "field effect" of this lesion for the development of invasive carcinoma [3-5]. With this new emerging evidence, there are increasing concerns regarding the biological significance of LCIS. The hypothesis is the presence of LCIS component associated with invasive carcinoma will increase the risk of local failure with breast conservation surgery, especially if LCIS is seen at the surgical margin. Since LIN at a margin is not mentioned routinely in pathology reports, it would be challenging to do any follow-up studies on the biological significance of LIN seen at margins. At our institution, however, margin assessment with LIN, both ALH and LCIS, has been routinely noted as the part of pathology report for the last 10 years. Re-excision was not mandatory if LIN was found at the surgical margins but left to each individual patient's decision based on a careful balanced discussion with the surgeon. Our hypothesis is that LIN is most likely not just a risk factor, but a precursor lesion to invasive cancer, and that LIN at the surgical margin may have a significantly higher recurrence rate over clear margins on BCT. The purpose of our study was to determine the recurrence rate of patients who had LIN at the margin after BCT at a single institution.

## Methods

**Figure 1:**
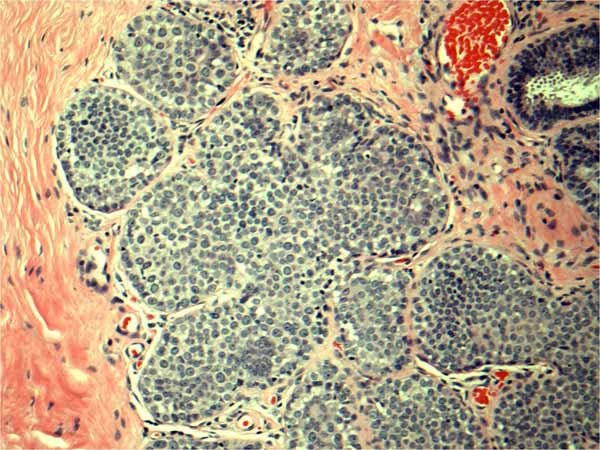
**Classic LCIS (LIN 1)**. H&E × 40 magnification: Classic type lobular carcinoma in situ (LCIS) with small monotonous dyshesive cells expanding terminal ductal lobular unit. LIN 1 by WHO criteria

**Figure 2:**
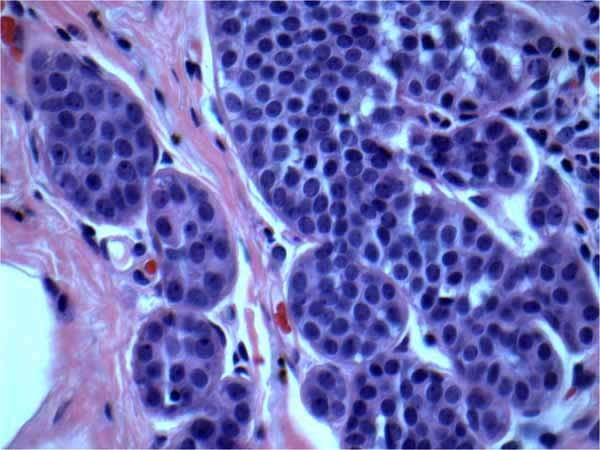
**Atypical lobular hyperplasia (AHL) (LIN 1)**. H&E × 60 magnification: Atypical lobular hyperplasia (ALH) shows similar nuclear features as classic LCIS but less expansion of terminal ductal lobular unit

**Figure 3:**
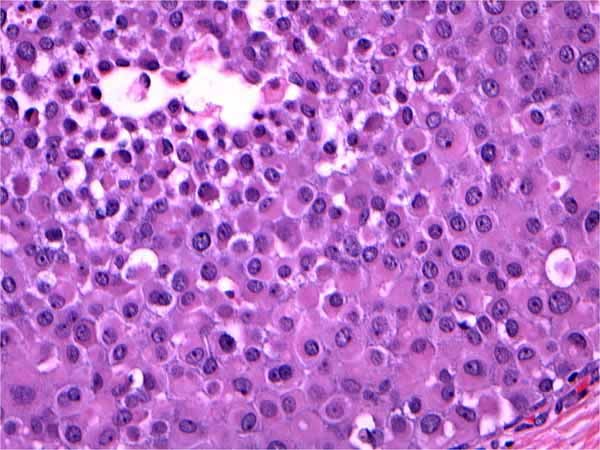
**Pleomorphic LCIS (LIN 3)**. H&E × 40 magnification: Pleomorphic LCIS exhibiting large, dyshesive, and apocrine-like cells. LIN 3 by WHO criteria

**Figure 4:**
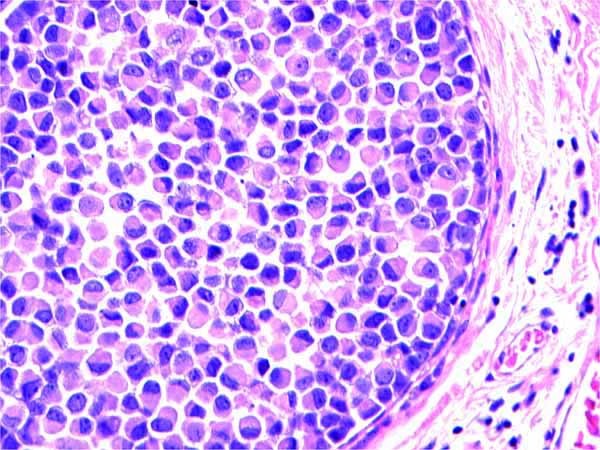
**Pleomorphic LCIS (LIN 3)**. H&E × 40 magnification: Pleomorphic LCIS exhibiting large, signet ring-like, dyshesive cells. LIN 3 by WHO criteria

**Figure 5:**
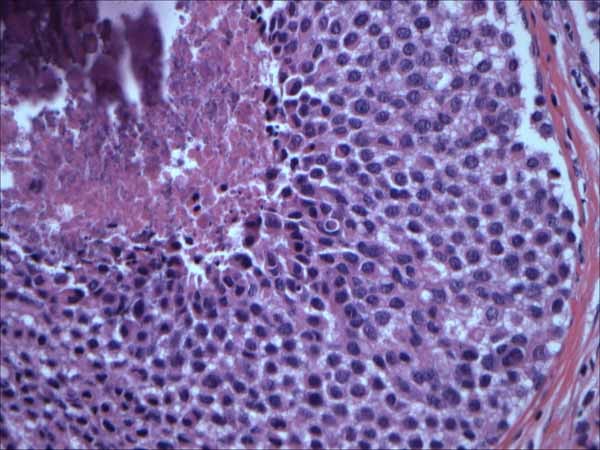
**Distended large cell LCIS with necrosis (LIN 2)**. H&E × 40 magnification: Distended large cell LCIS with central necrosis and calcifications. Nuclei are less pleomorphic and were considered LIN 2 by WHO criteria.

We retrospectively reviewed a total of 1,334 breast surgical excision specimens at a single institution in a 10 year period with a keyword search of "breast, lumpectomy, excision, lobular carcinoma in situ (LCIS) and atypical lobular hyperplasia (ALH)" via our Tamtron Powerpath computer system, after obtaining institutional review board (IRB) approval. We routinely did not use LIN terminology and WHO grading of LIN 1, 2 and 3 in our institution. Our terminology composed of ALH, and LCIS which were the classic type, LCIS with distension and comedo necrosis with nuclear grade 2 and pleomorphic LCIS with nuclear grade 3. Our terminology can be extracted, however, to WHO classifications: LIN grade 1 being ALH and LCIS classic type, LIN grade 2 being LCIS with distension and with or without comedo necrosis with nuclear grade 2 and LIN grade 3 being pleomorphic LCIS with nuclear grade 3. (See Figures 1, 2, 3, 4, 5, &6). Most of the cases of invasive carcinoma had mass lesion on mammogram, ultrasound and MRI. Most of the cases of pleomorphic LCIS and DCIS had pleomorphic calcifications on mammogram without mass lesions. Representative images are shown in Figures 7, 8, &9.

**Figure 6:**
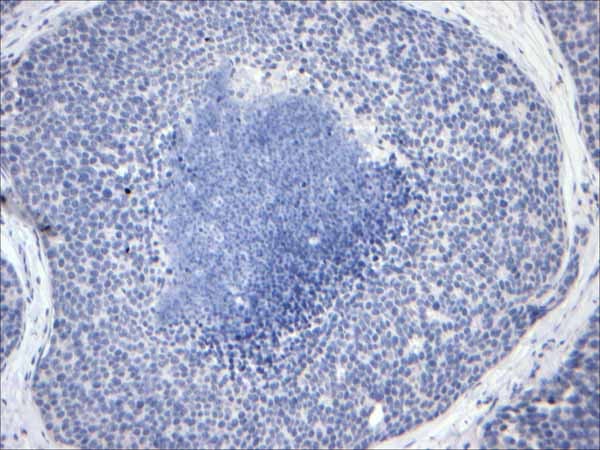
**E-cadherin stain**. E-cadherin immunohistochemical stain from the figure 5 is negative supportive of lobular differentiation.

**Figure 7:**
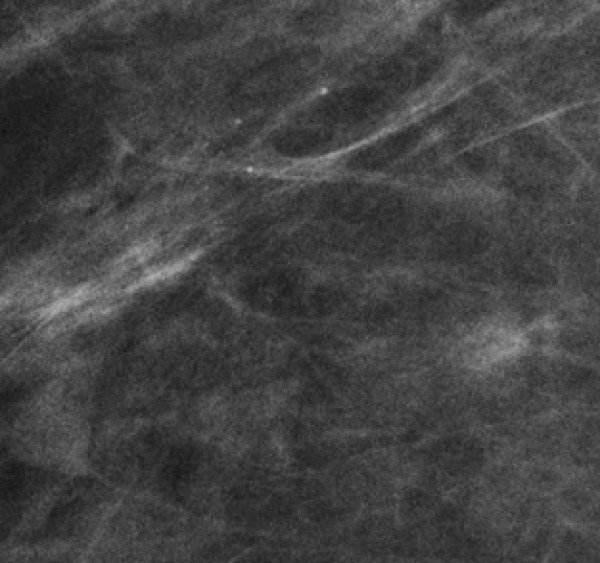
**Mammogram**. Magnified mediolateral view on mammogram shows clustered pleomorphic calcifications without associated mass, distortion, or other principal abnormality. Pathology showed pleomorphic lobular carcinoma in situ (pLCIS).

**Figure 8:**
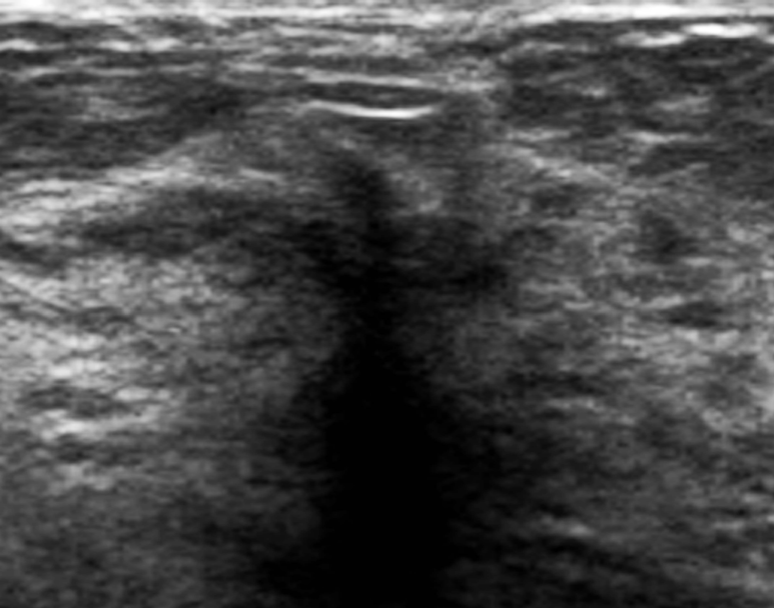
**Ultrasound**. Ultrasound demonstrates a vague area of focal shadowing and distortion without a discrete mass. Pathology showed invasive lobular carcinoma.

**Figure 9:**
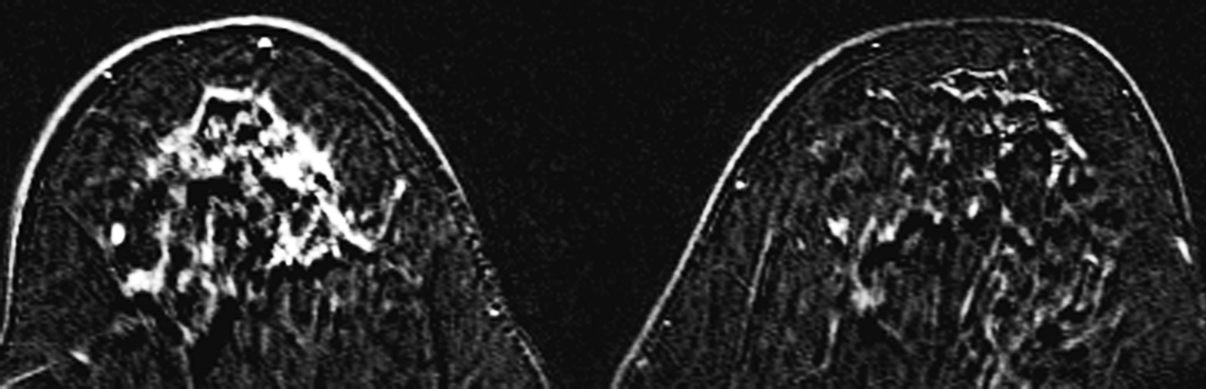
**MRI**. MRI demonstrates a large, irregular, enhancing mass with spiculated margins in the right breast. Pathology showed invasive lobular carcinoma.

A positive group was defined by patients who had a primary breast conservation surgery containing invasive mammary carcinoma, DCIS, or LIN, with either LCIS or ALH seen at the final margin. When LCIS or ALH was present in the specimen, it was defined as being present at the final margin if the lesion was described as touching an inked surface. Margins were defined as close for invasive carcinoma or DCIS if tumor cells were within 2 mm of an inked margin. We excluded all cases if invasive carcinoma or DCIS was found less than or equal to 2 mm from the final margin from the positive group. The primary endpoint of the study was local residual disease or recurrence in the ipsilateral breast. When re-excision showed residual invasive mammary carcinoma or DCIS or pleomorphic type LCIS, we defined the individual as positive for local recurrence. Patients who had negative findings, or residual classic LCIS or ALH were considered as a negative for local recurrence. To match the positive group, we selected 38 patients who had invasive mammary carcinoma or DCIS in addition to LIN, but margins negative for all lesions including LCIS/ALH. This sample of 38 patients was analyzed as a negative group. Available clinical follow-up was done for both positive and negative groups.

## Results

### Positive group

From a total of 1,334 breast surgical excision specimens, we identified 38 cases (2.8%) fitting our inclusion criteria for the positive group. Of the 38 cases: 5 (13%) were lost to follow-up, 12 (32%) had no further procedures performed and 21 (55%) had subsequent pathology specimens which consisted of 10 mastectomies, 10 re-lumpectomies, and 1 re-lumpectomy followed by mastectomy. Of 21 patients with re-excision, 12 (57%) had residual invasive carcinoma or DCIS, three (14%) had pleomorphic LCIS, 4 (19%) showed residual classic type LCIS, one (5%) had contralateral DCIS, and one (5%) had no residual tumor. Out of 21 patients who had re-excisions, 71% had significant residual disease (local recurrence), and 29% had no residual disease on re-excision. The clinical reasons that prompted re-excision in 21 patients with LCIS at the margin from the initial breast conservation surgery were not clearly documented in the medical record. Per surgeons, most of these patients decided to undergo additional surgeries mainly due to two factors; LCIS being a risk factor to developing into cancer in the future and any lesion being "positive" at the margin in the pathology report are enough to cause anxiety. Also, some of the patients who had invasive lobular carcinoma were not detected in a routine mammogram but additional imaging modality such as ultrasound and MRI were done to detect the presence of tumor. And hence, the annual clinical follow-up studies after BCT would be a financial burden to some patients. Thirteen (34%) cases had negative residual diseases or no recurrence in their clinical follow up. The median follow up from 12 patients who had no re-excision was 36 months [mean follow up of 40.1 months], ranging from 1 to 109 months with mammogram follow-up. Hormonal receptor studies were performed on invasive carcinoma or DCIS cases only. We did not perform hormonal receptors on classic LIN or pleomorphic LIN cases. However, most of the pleomorphic LIN with grade 3 cases had E-cadherin stain. See Additional file 1.

### Negative group

A negative control group consisted of matched 38 cases with BCT of invasive carcinoma and/or in-situ carcinoma that also had LIN in the specimen, but with clear margins. No patients had re-excision from this group. Both groups were matched to tumor type, stage and grade, lymph node status and patient age with clinical follow up in the patient's medical records. See Additional File 2.

From the negative control group, we found two (5.2%) patients with bone or brain metastasis and one (2.6%) with local recurrence. Follow up months ranged from 4 to 93 months, with a median of 47.5 months [mean of 45.6 months].

## Discussion

There is controversy in the management of the lobular intraepithelial neoplasia (LIN), namely atypical lobular hyperplasia (ALH)/lobular carcinoma in situ (LCIS) diagnosed by core needle biopsy without other proliferative lesions such as atypical ductal hyperplasia (ADH), ductal carcinoma in situ (DCIS), or invasive carcinomas. Some recommend excisional biopsy and others support no excision. There is consensus amongst pathologists, however, regarding the margin assessment of LIN on excisional biopsy; it is not mandatory to report LIN at the margin as in DCIS or invasive carcinoma and most pathologists do not report LIN at the margin. Obtaining a margin clear of LIN is not a surgical goal. Generally, the presence of LIN at the lumpectomy margin is regarded as irrelevant by most clinicians. The lack of consensus on this topic is illustrated by the results of a recent online survey by the American Society of Breast Disease. The survey posed the question of appropriate management of a patient with LCIS at the margin of a lumpectomy for invasive cancer. 40% of those who replied stated that they would consider re-excision in this circumstance and 8% stated that they would always perform a re-excision. (Observation from American Society of Breast Disease. ASBD Advisor 2008; Feb 17).

Also, unlike DCIS, patients with LIN alone on excision are not treated with radiation after breast conservation therapy unless there is a concomitant DCIS or invasive carcinoma. With emerging new evidence that LIN is not just a risk factor but a precursor lesion to invasive carcinoma, margin status may play an important role in the treatment of patients with LIN. If LIN is indeed a precursor lesion, then its presence at the surgical margin in a lumpectomy specimen should increase local recurrence in patients treated with breast-conserving therapy. In an attempt to test this hypothesis, we compared local residual/recurrence rates in patients with and without LIN at the margin who underwent breast conservation surgery for the treatment of invasive carcinoma or DCIS. Ideally, the positive and negative control groups should have a similar disease process except for margin status. The positive group included some cases that had no cancer/DCIS, i.e. only LCIS, with LCIS/ALH at the margin but the negative group did not have any cases that had only LCIS/ALH in the specimen. This was done so that our study would underestimate rather than possibly overestimate the risk of having LIN at the margin, especially given the fact that it is clinically a short-term follow-up.

As it is often difficult to differentiate between a local recurrence and a new primary in the treated breast, all tumor found in the ipsilateral breast during follow-up were classified as local recurrences in our study. Technically it shouldn't be considered as recurrences, but considered as residual tumor because the cancer was probably there at the time of initial resection, and not progressed from the margin LIN.

Our study showed 39% had significant recurrent diseases when LIN was seen at the surgical margin of initial breast conservation therapy, compared to 7.9% from negative control group. This 7.9% includes patients with metastasis in the negative group, but the 39% from positive group counts only local recurrence.

There are limited numbers of LIN recurrence data after only breast conservation therapy. The reported frequencies of recurrence of invasive carcinoma after LCIS diagnosis is approximately 10% and 20% of patients at 10 years and 20 years, respectively. The survival rate at 15 years was 100% in a cohort of 32 patients [6-8].

The NSABP B-17 study analyzed a subset of 182 patients with LCIS in addition to DCIS and treated with breast conservation therapy alone was compared to DCIS alone in the incidence of recurrence. The ipsilateral recurrence of LCIS and DCIS was significantly lower than DCIS alone; 2.2% in LCIS and DCIS and 12% in the DCIS alone respectively. Also, the contralateral breast recurrence incidence was 1.1% compared to 9% respectively. Their conclusion was that the presence of LCIS should not lead to more surgery such as mastectomy [9].

A similar conclusion was drawn from Carolin K et al. [10] study which compared LCIS and invasive cancer group to invasive cancer without LCIS; there was no significant increase in ipsilateral or contralateral breast recurrence in a total of 105 patients with LCIS and 115 patients without LCIS. Over time, there was no increase in relapse noted for the patients who had LCIS as a histologic component of invasive carcinoma.

Jobsen et al. [11] looked at the impact of margin status and outcome of invasive lobular carcinoma treated with breast-conserving therapy and followed the long term outcome from a single region in the Netherlands during the last twenty years with 318 patients and 33 re-excision. They found that the positive margins for ILC seem to be a strong predictor for local recurrence in women less than or equal to 50 years of age but the distant metastasis free survival and disease free survival were not affected by the margin status. LCIS alone did not show significance in relation to local control. Their study showed a trend towards an increased local recurrence rate with positive margins for LCIS only and this seems limited to women greater than 50-years of age.

Robin M. Ciocca et al. [12] studied whether or not LCIS at the margin would increase local recurrence in patients treated with breast-conserving therapy. In their 84 patients with LCIS present at the specimen margin, the crude rate of local recurrence for patients with and without LCIS was 4.5% and 3.8% respectively. They concluded that re-excision is not indicated even if LCIS is present at the margin.

Jolly et al., [13] on the contrary, found that the presence of LCIS was associated with a higher incidence ipsilateral recurrence; 14% with LCIS at the margin when compared to 7% without LCIS at the margin from a patient sample of 56 with a median of 8.7 years follow up.

Stolier A et al. [14] reported no local recurrence with LCIS in 40 patients with 38% involved or close margin with LCIS in 67 months follow-up period. Ben-David M et al. [15] reported that the presence of LCIS at the margins and the multifocal extent of LCIS did not alter the rate of local recurrence in their 64 patients' samples who received breast conservation and radiation treatment with the median follow-up time of 3.9 years. Abner et al. [16] reported similar results; that the extent of LCIS and positive margin with LCIS did not have increased local recurrence rate in 8 years. Sasson et al. [17] reported that the ipsilateral local recurrence rate of 29% in the LCIS group as opposed to 6% in the generally treated population. However, when tamoxifen treatment was used as hormonal therapy, the difference was not significant; 8% as opposed to 6%, comparing the LCIS group to the control group.

Literature supports that with the incidences of ipsilateral and contralateral recurrence rates, mortality rates are low with LIN, and even with invasive lobular carcinoma. This may be due to the fact that the development of invasive carcinoma after LIN takes a long time. Most of the studies have a short median follow-up and cannot exclude the possibility that, with a longer follow-up duration, an impact of LIN on local recurrence will be significantly increased. In fact, the study of Rosen et al. reported the average interval to the development of cancer was 20.4 years after biopsy [18]. Another study by Page et al. [19] showed 75% of cancers developed within 15 years of biopsy of LCIS.

Long term follow-up of LIN treated with BCT alone has demonstrated a 1% per year cumulative long-term risk of breast carcinoma persisting even 10-20 years after diagnosis. Like most retrospective studies, the inability to prospectively evaluate the conclusions and hypotheses is a limiting factor, as well as a short term follow up in LIN. Clinical follow-up of more than 5 years is needed to verify the significance of LIN in the ipsilateral recurrence. Prospective randomized trials related to therapy of patients with LIN at the margin are needed to clearly understand the risk of local recurrence. However, a low mortality rate supports the view that mastectomy is not indicated to clear LIN at the margin if LIN is classic type and grade 1. If there is an overlapping feature of LCIS with DCIS, then re-excision is not unreasonable.

There is no doubt that LIN is a risk factor for subsequent carcinoma, and morphologic, immunohistochemical and epidemiologic observations support the statement that LIN is also a direct precursor to invasive carcinoma. Although our data is limited due to the fact that it is a retrospective study and small in sample size, such data would only add to the limited number of LIN recurrence rate after breast conservation therapy. The limitation of our study is that there may be follow-up bias. In our negative control group, there are not any additional surgeries so we cannot be sure that there are no cancerous lesions in the control group.

## Conclusions

In order to better understand the local recurrence rate to ipsilateral, contralateral, and systemic metastatic rate, it would be important to include the description of margin status by LIN. Our data suggests that the adequacy of the excision with LIN at the margin should be considered, and re-excision is recommended to decrease recurrence rate (significant residual disease).

## Competing interests

The authors declare that they have no competing interests.

## Authors' contributions

SKA has made contributions to conception, design, interpretation of data, drafting the manuscript, and approval of the final version of the paper. MM carried out the acquisition of data and contributed important intellectual content. EPO carried out participation in the design of the study and performed analysis of data. NAM carried out drafting and revising the paper critically. All authors read and approved the final manuscript.

## Supplementary Material

Additional file 1 **Diagnostic pathology**. LCIS Table S1. Click here for file

Additional file 2 Table S2: Diag pathol. Click here for file
