# Supplementary material for: Significance of lobular intraepithelial neoplasia at margins of breast conservation specimens: a report of 38 cases and literature review
Source: Diagn Pathol. 2010 Aug 20;5:54. doi: 10.1186/1746-1596-5-54 (PMC2936385; doi:10.1186/1746-1596-5-54)
Supplement: Additional file 1 — Diagnostic pathology. LCIS Table S1. [file 1746-1596-5-54-S1.DOC]

Table 1: Positive control group: LCIS seen at surgical margin

| No. | Age | BCS final Dx | ER/PR | E-cadherin | Tumor  stage | LN | Grade | Re-ex/Mast. | Re-ex/Mast. Dx. | Recurrence | Surg f/u  (mos) | Mammo f/u  (mos) |
| --- | --- | --- | --- | --- | --- | --- | --- | --- | --- | --- | --- | --- |
| 1 | 47 | ILC, DCIS, LCIS | +/+ | NA | T1 | 0/3 | G2 | Mast | 1mm ILC. LCIS | + | 2 | NA |
| 2 | 58 | ILC, pLCIS | +/+ | - | T1 | 0/2 | G2 | Mast | pLCIS | + | 2 | NA |
| 3 | 61 | ILC, pLCIS | +/+ | - | T1 | ITC/1 | G2 | Re-ex and Mast | 1mm ILC and pLCIS 5.5 cm | + | 4 | NA |
| 4 | 45 | IDC, DCIS, LCIS | +/+ | NA | T1 | 0/1 | G1 | Re-ex | DCIS, ALH | + |  | 42 |
| 5 | 47 | IDC, LCIS | +/+ | NA | T1 | 0/3 | G1 | Re-ex | 1.8 cm ILC + 0.4 cm LCIS | + | 1 | 35 |
| 6 | 45 | ILC, pLCIS | +/+ | - | T1 | 0/4 | G1 | Re-ex | pLCIS | + | 1 | 36 |
| 7 | 64 | ILC, DCIS | +/- | NA | T2 (4cm) | 0/26 | G3 | Mast | 6cm ILC | + | 44 | NA |
| 8 | 68 | DCIS, LCIS | -/- | NA | Tis | NA | G3 | Ipsilateral Re-ex  Contralateral ex | contralateral DCIS | + | 49 | NA |
| 9 | 55 | ILC, LCIS | +/+ | NA | T2 | 0/33 | G2 | Mast | 2 cm ILC, LCIS | + | 4 | NA |
| 10 | 52 | pILC, pLCIS | -/- | - | T1 | 2/9 | G3 | Re-ex | 1 cm pLCIS, pLCIS | + | <1 | - |
| 11 | 52 | ILC, LCIS | +/+ | NA | T2 | 0/3 | G2 | Re-ex | 2mm ILC | + | <1 | - |
| 12 | 47 | pILC, PLCIS | +/- | - | T1 | 0/13 | G3 | Re-ex | 0.7 cm pILC, pLCIS | + | 1 | - |
| 13 | 54 | ILC, LCIS | +/+ | NA | T1 | 0/15 | G1 | Mast/bil | DCIS, LCIS, ADH | + | 5 | NA |
| 14 | 45 | ILC, DCIS | +/+ | NA | T1 | 0/2 | G1 | Re-ex | DCIS, LCIS | + | 1 | - |
| 15 | 47 | DCIS, LCIS | -/- | NA | Tis | 0/14 | G3 DCIS | Mast | DCIS ext LCIS classic | + | 3 | NA |
| 16 | 44 | ILC, LCIS | +/+ | NA | T1 | 0/1 | G2 | Mast | 1.7 cm ILC, LCIS | + | 63 | 63 |
| 17 | 50 | LCIS, ALH | NA | NA | Tis | NA | classic | Mast/bil | LCIS, ALH | - | 2 | NA |
| 18 | 58 | ILC, LCIS | +/+ | NA | T1 | 0/1 | G2 | Re-ex | Ext classic LCIS | - | 2 | 8 |
| 19 | 80 | ILC, LCIS | +/+ | NA | T1 | 1/4 | G2 | Re-ex | No tumor | - | 2 | 11 |
| 20 | 43 | ILC LCIS | +/+ | NA | T1 | 0/2 | G1 | Mast/bil | Ext classic LCIS | - | 3 | NA |
| 21 | 77 | ILC, signet LCIS | +/+ | - | T2 | 0/1 | G2 | Mast/bil | LCIS | - | 4 | NA |
| 22 | 61 | DCIS, LCIS | +/+ | NA | Tis | NA | G1 | - | NA | - | No | 49 |
| 23 | 53 | LCIS, ALH | NA | NA | Tis | NA | classic | - |  | - | No | 14 |
| 24  Table 1: Positive control group: LCIS seen at surgical margin (cont.) | 47 | LCIS, ALH | NA | NA | Tis | NA | classic | - | NA | - |  | 23 |
| 25 | 41 | LCIS | NA | NA | Tis | NA | classic | - | NA | - |  | 10 |
| 26 | 65 | DCIS, LCIS | +/+ | NA | Tis | NA | classic | - | NA | - |  | 16 |
| 27 | 58 | LCIS, ALH | NA | NA | Tis | NA | classic | - | NA | - |  | 33 |
| 28 | 43 | pLCIS | NA | - | Tis | NA | G3 | - | NA | - |  | 30 |
| 29 | 68 | ILC, DCIS, LCIS | +/+ | NA | T2 | 0/3 | G1 | - | NA | - |  | 50 |
| 30 | 66 | ILC, signet LCIS | +/- | - | T1 | 1/10 | G2 | - | NA | - |  | 39 |
| 31 | 62 | ILC, LCIS | +/+ | NA | T1 | ITC/2 | G2 | - | NA | - |  | 58 |
| 32 | 68 | ILC, DCIS, LCIS | +/+ | NA | T2 | 0/3 | G1 | - | NA | - |  | 50 |
| 33 | 63 | IDC, DCIS, LCIS | +/- | NA | T1 | 1/12 | G2 | - | NA | - |  | 109 |
| 34 | 59 | ILC, LCIS | +/+ | NA | T1 | 0/2 | G1 | lost | NA | Lost | Lost | Lost |
| 35 | 73 | LCIS | NA | NA | Tis | NA | G1 | lost | NA | Lost | lost | Lost |
| 36 | 44 | pLCIS | NA | - | Tis | NA | G3 | lost | NA | Lost | Lost | Lost |
| 37 | 47 | LCIS | NA | NA | Tis | NA | G1 | lost | NA | Lost | Lost | Lost |
| 38 | 40 | ILC, LCIS | +/+ | NA | T2 | 2/12 | G2 | lost | NA | Lost | Lost | Lost |

Table 2: Negative control group; no LCIS seen at surgical margin (cont.)
