# Supplementary material for: Significance of lobular intraepithelial neoplasia at margins of breast conservation specimens: a report of 38 cases and literature review
Source: Diagn Pathol. 2010 Aug 20;5:54. doi: 10.1186/1746-1596-5-54 (PMC2936385; doi:10.1186/1746-1596-5-54)
Supplement: Additional file 2 — Table S2: Diag pathol. [file 1746-1596-5-54-S2.DOC]

Table 2: Negative control group; no LCIS seen at surgical margin

| No | Age | Final Dx | ER/PR | E-cadherin | tumor stage | LN status | Grade | Recurrence | F/u mos |
| --- | --- | --- | --- | --- | --- | --- | --- | --- | --- |
| 1 | 63 | ILC, LCIS | +/+ | NA | T2 | 0/2 | G1 | - | 33 |
| 2 | 71 | ILC, LCIS | +/+ | NA | T2 | 0/2 | G2 | - | 78 |
| 3 | 71 | ILC, LCIS | +/+ | NA | T2 | 10/14 | G2 | mets bone, brain | 76 |
| 4 | 44 | ILC, LCIS, DCIS | +/+ | NA | T3 | 3/15 | G2 | - | 5 |
| 5 | 71 | ILC, DCIS, LCIS | +/+ | NA | T2 | 1/6 | G1 | - | 59 |
| 6 | 56 | IDC, DCIS, LCIS | +/+ | NA | T1 | 0/8 | G1 | - | 28 |
| 7 | 44 | IDC, DCIS, ALH | +/+ | NA | T2 | 0/9 | G2 | - | 78 |
| 8 | 34 | IDC, LCIS, ALH | +/+ | NA | T1 | 0/39 | G1 | bone mets | 23 |
| 9 | 50 | IDC, DCIS, LCIS | +/+ | NA | T1 | 0/1 | G1 | - | 28 |
| 10 | 50 | IDC, DCIS, LCIS | +/+ | NA | T1 | 0/1 | G1 | - | 62 |
| 11 | 53 | IDC, DCIS, LCIS | +/+ | NA | T2 | 3/15 | G2 | - | 58 |
| 12 | 77 | ILC, DCIS, LCIS | +/+ | NA | T1 | 0/1 | G1 | - | 73 |
| 13 | 54 | ILC, DCIS, LCIS | +/+ | NA | T1 | NA | G2 | - | 70 |
| 14 | 58 | DCIS, LCIS | +/+ | NA | Tis | NA | G1 | - | 43 |
| 15 | 87 | IDC, DCIS, LCIS | +/+ | NA | T1 | 0/5 | G1 | - | 18 |
| 16 | 66 | DCIS, LCIS | +/- | NA | Tis | NA | G3 | - | 68 |
| 17 | 54 | DICS, LCIS | -/- | NA | Tis | 0/2 | G3 | - | 55 |
| 18 | 50 | IDC, DCIS, LCIS | +/+ | NA | T1 | 0/3 | G1 | - | 54 |
| 19 | 60 | IDC, DCIS, LCIS | +/+ | NA | T2 | ITC/5 | G2 | - | 66 |
| 20 | 52 | IDC, DICS, LCIS | +/+ | NA | T2 | 1/37 | G1 | - | 57 |
| 21 | 58 | IDC, DCIS, LCIS | -/- | NA | T3 | 0/3 | G3 | - | 48 |
| 22 | 50 | IDC, DCIS, LCIS | +/+ | NA | T1 | 0/3 | G1 | - | 63 |
| 23 | 82 | IDC, DCIS, LCIS | +/- | NA | T2 | 0/4 | G3 | - | 44 |
| 24 | 73 | IDC, DCIS, LCIS | +/+ | NA | T1 | 1/1 | G3 | - | 5 |
| 25 | 49 | IDC, DCIS, ALH | +/+ | NA | T1 | 0/3 | G1 | - | 41 |
| 26 | 52 | IDC, DCIS ALH | +/+ | NA | T2 | 0/1 | G2 | - | 47 |
| 27 | 65 | IDC, DCIS, LCIS | +/+ | NA | T2 | 0/17 | G2 | - | 54 |
| 28 | 44 | IDC, DCIS, LCIS | +/+ | NA | T2 | 0/6 | G1 | - | 50 |
| 29 | 49 | IDC, DCIS, LCIS | +/+ | NA | T1 | 0/1 | G1 | recur | 93 |
| 30 | 47 | DCIS, LCIS | +/+ | NA | Tis | NA | G2 | - | 50 |
| 31 | 65 | IDC, LCIS, ALH | +/+ | NA | T3 | 1/3 | G1 | - | 24 |
| 32 | 51 | DCIS, LCIS | -/- | NA | Tis | NA | G3 | - | 4 |
| 33 | 63 | IDC, DCIS, LCIS | +/+ | NA | T2 | 1/7 | G2 | - | 17 |
| 34 | 85 | IDC, LCIS | +/+ | NA | T1 | 0/4 | G1 | - | 23 |
| 35 | 49 | IDC, DCIS, ALH | +/+ | NA | T1 | 1/3 | G3 | - | 29 |
| 36 | 80 | IDC, DCIS, LCIS | +/+ | NA | T1 | NA | G1 | - | 36 |
| 37 | 59 | ILC, LCIS | +/+ | NA | T1 | 0/3 | G2 | - | 36 |
| 38 | 46 | IDC, DCIS, ALH | +/+ | NA | T1 | 0/1 | G2 | - | 38 |

BCS: Breast conservation surgery, Dx: Diagnosis, ILC: invasive lobular carcinoma, pILC: pleomorphic invasive lobular carcinoma, LCIS/ALH: lobular carcinoma in situ/atypical lobular hyperplasia, pLCIS: pleomorphic lobular carcinoma in situ, DCIS: ductal carcinoma in situ, ER: Estrogen receptor, PR: Progesterone receptor, NA: not applicable Mast/bil: Mastectomy/ bilateral. Ext: Extensive, Re-ex: Re-excision, Mammo: Mammogram, f/u: follow-up,-: negative, +: positive, G1: Grade 1, well differentiated, G2: Grade 2, moderately differentiated, G3: Grade 3, poorly differentiated, mos.: months

Table 2: Negative control group; no LCIS seen at surgical margin (cont.)
